# Transcavernous Approach to the Basilar Apex: A Cadaveric Prosection

**DOI:** 10.7759/cureus.2192

**Published:** 2018-02-14

**Authors:** Jonathan N Sellin, Visish M Srinivasan, Jovany C Navarro, Hunt H Batjer, Harry Van Loveren, Edward A Duckworth

**Affiliations:** 1 Department of Neurosurgery, Baylor College of Medicine; 2 Department of Anesthesiology, Department of Anesthesiology and Critical Care, University of Pennsylvania, Philadelphia, PA; 3 Department of Neurosurgery, UT Southwestern Medical Center, Dallas, TX; 4 Department of Neurosurgery, University of South Florida Morsani College of Medicine; 5 Department of Neurosurgery, St. Lukes Regional Medical Center

**Keywords:** transcavernous approach, basilar apex, cadaveric prosection, anatomic study, aneurysm, basilar tip

## Abstract

The transcavernous approach to the basilar artery, as initially described by Dolenc, is one of the most common and elegant approaches to the region. It affords a generous working and viewing angle, but it can be technically challenging and requires attention to detail at each step. We investigate this approach in this report via a cadaveric prosection with a focus on the value of each of the component steps in improving surgical view and exposure. The transcavernous approach steps are divided into extradural stages: orbitozygomatic osteotomy (a modern adjunct to Dolenc’s original description), drilling of the lesser sphenoid wing, and anterior clinoidectomy; and intradural stages: wide splitting of the Sylvian fissure, unroofing of the oculomotor and trochlear nerves, and posterior clinoidectomy. The surgical windows afforded by each step in the approach are illustrated using microscopic images taken during the cadaveric prosection of a donor who happened to harbor a basilar apex aneurysm. An illustrative case and artist illustrations are used to emphasize the relative value of each step of the transcavernous exposure.

## Introduction

The upper retroclival region is a challenging anatomical region, affording only limited natural corridors: between the clivus anteriorly, the petrous apex laterally, and the brainstem posteriorly. These narrow confines are further complicated by the critical neurovascular structures that span this space. Given the nature of the pathology typical for the region—most commonly skull base tumors and basilar apex aneurysms—ideal surgical approaches must maximize viewing and working angles while limiting brain retraction. The advent of endovascular treatment for posterior circulation aneurysms has lowered the frequency with which neurosurgeons approach this region surgically. However, despite claims to the contrary, not all posterior circulation aneurysms can or should be treated endovascularly [1, 2]. Surgery, moreover, remains the gold standard treatment for symptomatic tumors in this region. Clearly, cranial neurosurgeons must maintain competency in approaching this region.

Numerous different approaches have been used to access this challenging area with variable outcomes [3-9]. During the heyday of skull base technique development, Dolenc described a novel approach to the region: *the transcavernous approach* [3]. Although the outcomes in his initial series would be considered suboptimal by today’s standards, the work was revolutionary nonetheless, and refinements to this technique have led to numerous reports of its successful application. It has emerged as an appealing approach for the surgical treatment of basilar apex aneurysms [1, 10].

In a seminal cadaveric prosection in 1991, Dolenc demonstrated his method of exposing the cavernous sinus [11]. The current study seeks to emulate that prosection and examine Dolenc’s transcavernous approach to the upper retroclival space. We perform a detailed technical review and an assessment of the contribution of each step of dissection to the final surgical view achieved by the approach.

## Technical report

Material and methods

Cadaver Preparation

**Figure 1:**
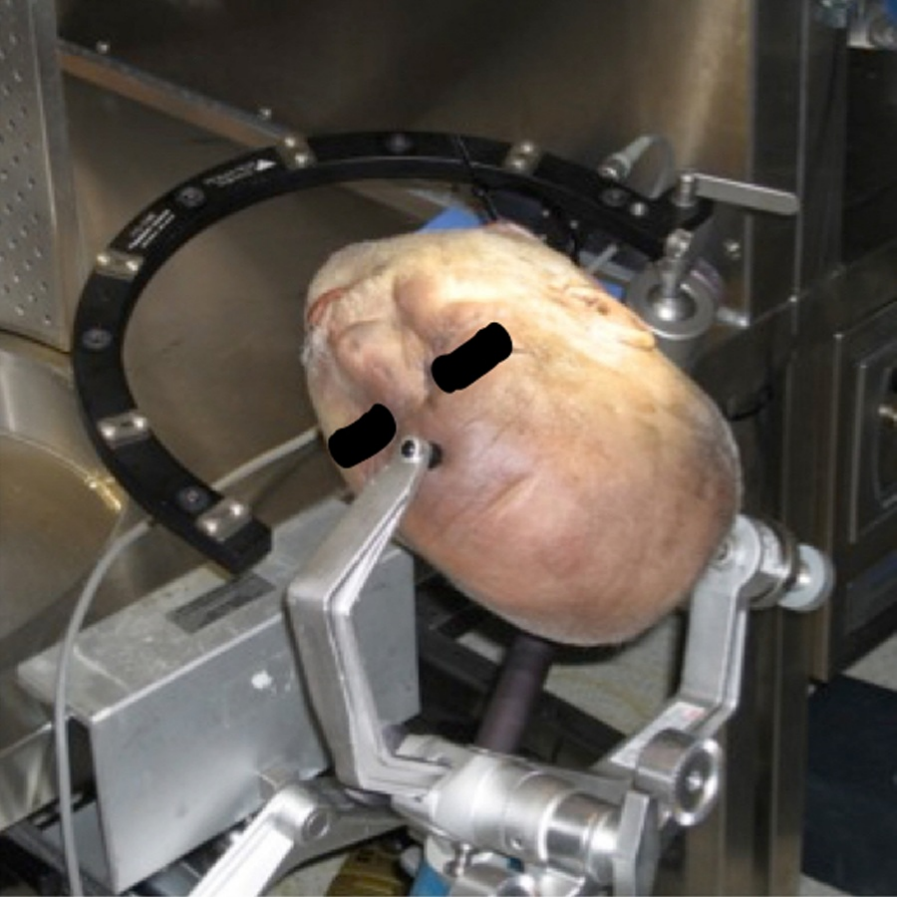
Head positioning in cadaveric prosection Cadaveric head positioned in Mayfield head holder, with associated stereotactic image guidance frame.

A laboratory prosection was carried out on an embalmed, alcohol-preserved cadaveric head, which happened to harbor a basilar tip aneurysm. A standard preparation of the head was performed: the internal carotid arteries and jugular veins were cannulated and irrigated with saline, then injected with red and blue-pigmented barium impregnated latex. A pre-dissection thin cut computed tomography (CT) scan (essentially a CT angiogram due to the presence of barium impregnated latex in the vasculature) was performed and the data used for frameless stereotaxic image guidance using a Medtronic StealthStation system (Medtronic, Minnesota, USA). The head was affixed in a Mayfield headholder (Integra Neurosciences, NJ, USA) in a position consistent with what is used in the operating room (Figure 1). Dissection was carried out using standard microsurgical instruments and a Zeiss Contraves operative microscope (Carl Zeiss, GmbH, Jena, Germany). Stepwise exposure was carried out starting deep and working superficially with photography using the operative microscope.

Dissection technique - prosection

**Figure 2:**
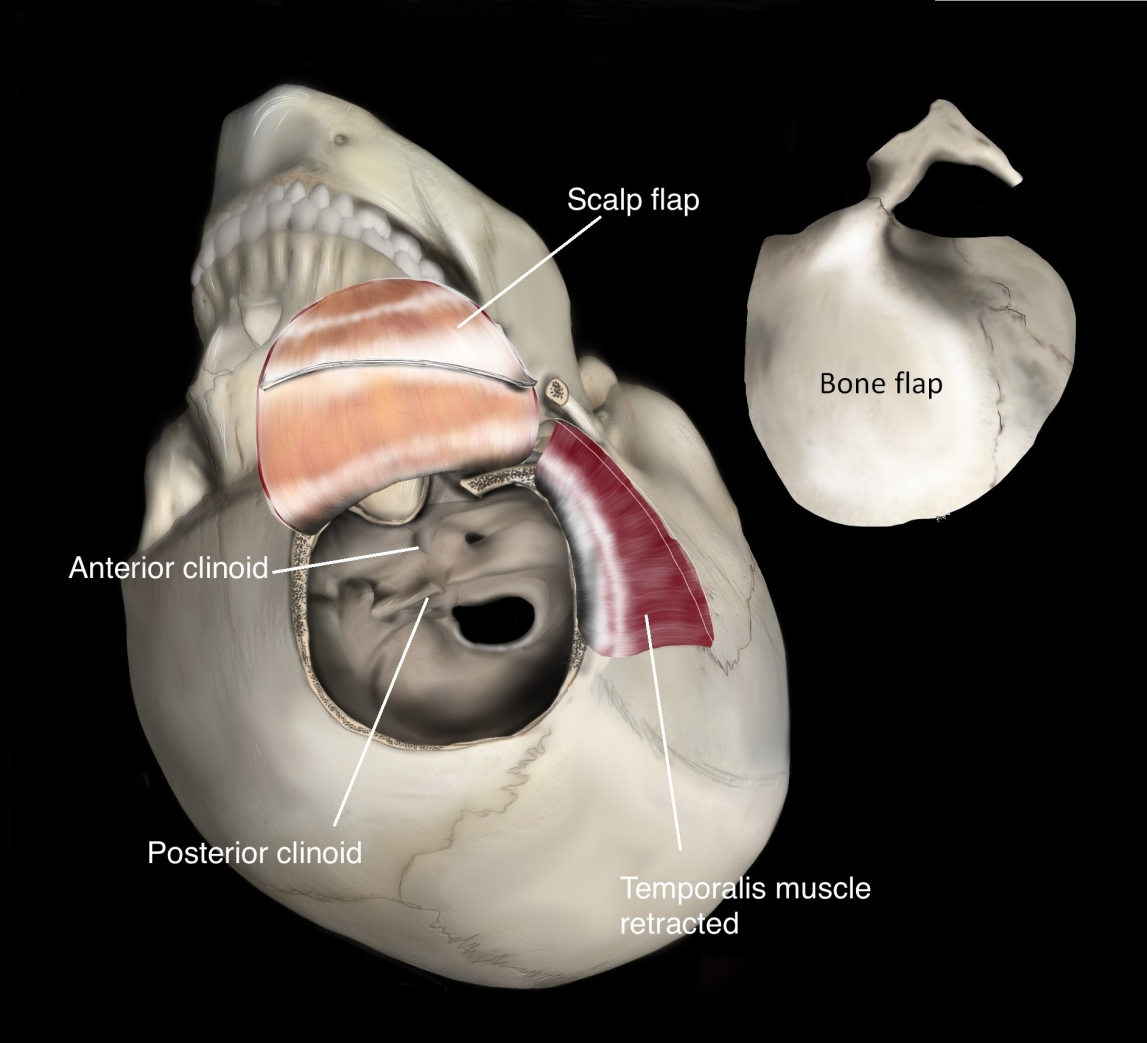
Artist's rendering of the orbitozygomatic craniotomy Artist’s illustration of a typical interfascial temporalis dissection and a “one-piece” frontotemporal orbitozygomatic (FTOZ) craniotomy. In our cadaveric experiment, the orbitozygomatic osteotomy was performed in a second step in order to understand the relative value of the additional bony exposure.

**Figure 3:**
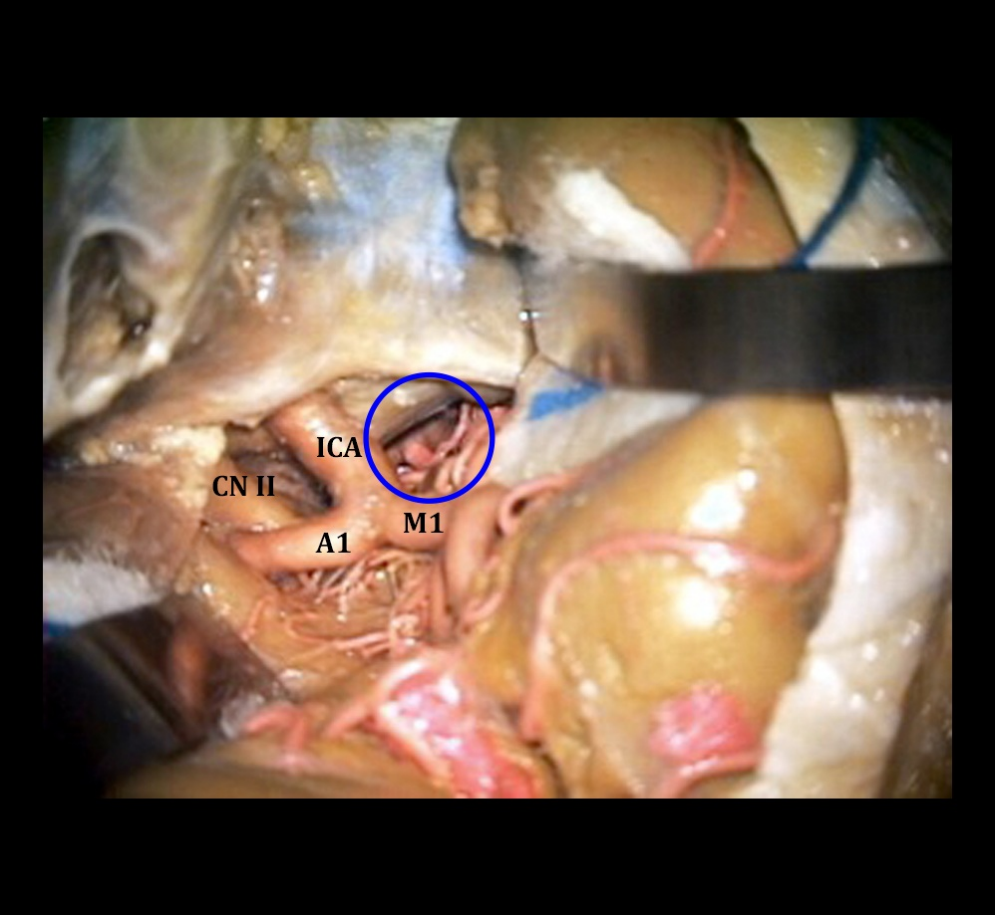
Exposure via the Sylvian fissure Cadaveric exposure of right intradural ICA after right-sided Sylvian fissure splitting, with the fortuitous small basilar apex aneurysm seen in the depths of the exposure. Orienting structures are labeled: internal carotid artery (ICA), optic nerve (CN II), middle cerebral artery (M1), and anterior cerebral artery (A1). The blue circle indicates the region of interest - deep structures include the basilar apex, posterior cerebral arteries, and a basilar apex aneurysm.

**Figure 4:**
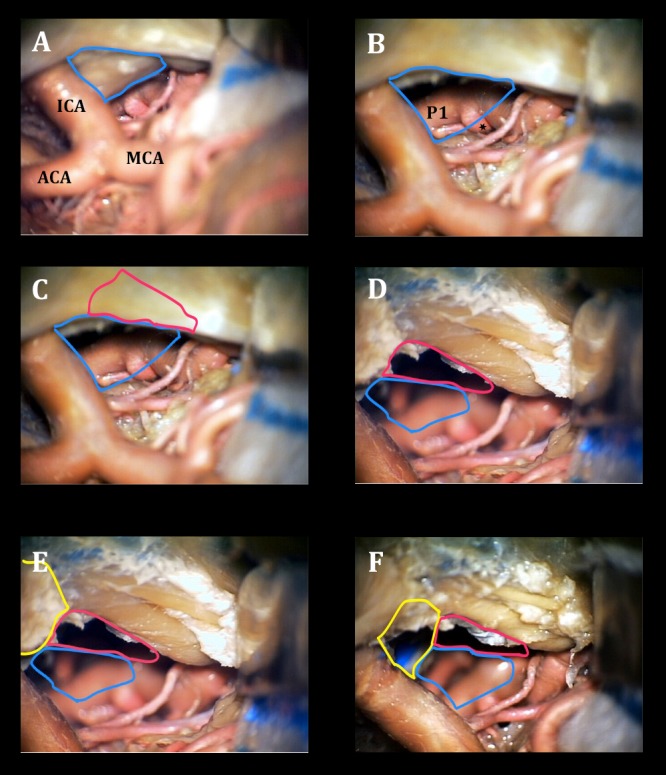
Step-wise exposure gained from the transcavernous approach Cadaveric prosection with view of the basilar apex via the right trans-sylvian view A) before and B) after posterior clinoidectomy (posterior clinoid process outlined in blue, aneurysm marked with a star); C) before and D) after cavernous sinus unroofing (space afforded by CS unroofing outlined in purple) E) before and F) after anterior clinoidectomy (space afforded by anterior clinoidectomy outlined in yellow). Orienting structures include the internal carotid artery (ICA), anterior cerebral artery (ACA), middle cerebral artery (MCA), posterior cerebral artery (P1). CS - cavernous sinus.

A standard frontotemporal scalp incision was made, extending from the root of the zygoma, anterior to the tragus, to the hairline at the midline. An interfascial dissection was performed, allowing for reflection of the scalp anteriorly and inferiorly, and the temporalis muscle posteriorly and inferiorly. In our cadaver, initially a pterional craniotomy was performed and later supplemented with an orbitozygomatic osteotomy as a final step (shown in our standard “one-piece” form in Figure 2). For the cadaver prosection, critical steps of the dissection were performed “in reverse” (deep to superficial) for the purpose of better understanding their relative value in increasing visualization. A curved dural incision was reflected towards the sphenoid wing to expose the Sylvian fissure (Figure 3) and the following steps were performed in sequence: posterior clinoidectomy, cavernous sinus unroofing, anterior clinoidectomy. The increasing visualization of the basilar apex following these steps in our cadaver prosection can be appreciated in Figure 4.

Modern Surgical Technique - Transcavernous Approach

Although for our prosection the dissection steps were performed “in reverse” for better understanding their relative value in increasing visualization, listed below are the steps of a more modern, updated version of the transcavernous approach, as they would be performed surgically.

Extradural Stages

**Figure 5:**
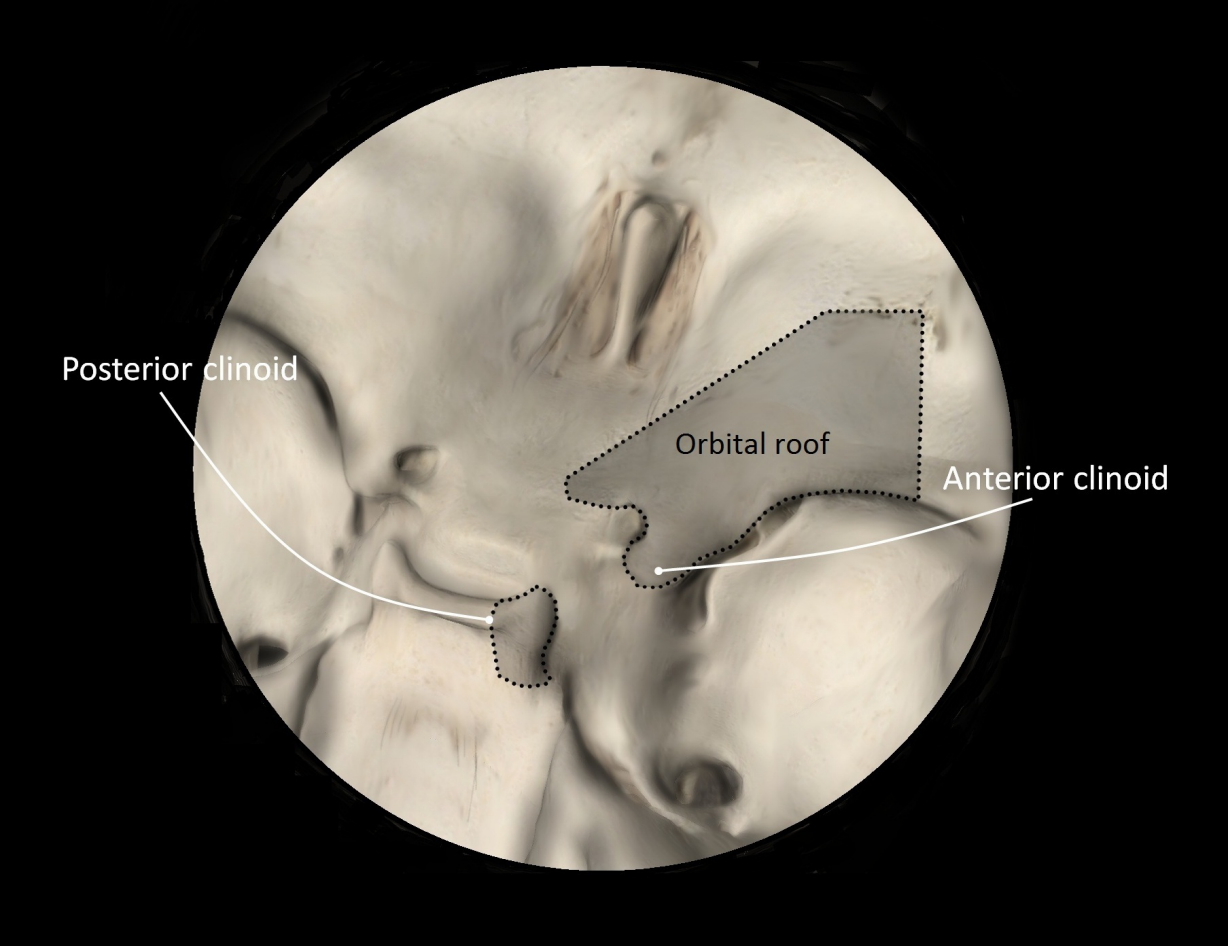
Osseous structures to be drilled down or removed An artist's rendering of anterior and posterior clinoidectomy.

First a frontotemporal orbitozygomatic (FTOZ) craniotomy is completed, in one or two pieces, as has been previously described [5, 12]. It is not always completely necessary to do a full OZ for the transcavernous approach, though it is common practice. Subsequently, a high-speed drill is used to remove critical boney structures prior to dural opening. First, the lesser sphenoid wing is aggressively drilled down until a flush “wall” is created, exposing the dura mater to the depth of the meningo-orbital ligament as it passes from the superior orbital fissure to the orbit (Figure 5). Additional drilling may also be performed along the remaining orbital roof and lateral orbital rim.

Next, an anterior clinoidectomy is completed, preferably extradurally. This is performed by first decompressing the optic nerve as the medial, superior, and lateral aspects of the optic canal are unroofed. Care is taken when drilling the medial wall of the optic canal not to open the lateral ethmoid sinuses. Finally, removal of the bone situated between the clinoidal segment of the internal carotid artery (ICA) and the medial wall of the optic canal—the optic strut—is done, allowing for gentle en bloc removal of the now disengaged anterior clinoid process (ACP) (Figure 5). Alternatively, the anterior clinoidectomy can be deferred and performed intradurally.

Although not performed in our prosection, extradural dissection of the cavernous sinus can further extend exposure. This additional step is relatively simple, safe, and offers several advantages. It effectively brings the ACP more superficial in the field, facilitating the ease of its removal. Second, it allows for injection of fibrin glue into the cavernous sinus between V1 and V2 [13]. Third, it improves the view of cranial nerve (CN) IV crossing over CN III, facilitating dissection along the oculomotor corridor posteriorly.

Dural Opening

Having completed all the extradural bone work, a standard curved dural incision is performed and the dura reflected anterolaterally along the globe. Taut tacking sutures are used, in fact, to retract the globe to a small degree using the dura. This may be further facilitated by additional removal of the orbital rom or roof, as needed.

Intradural Stages

**Figure 6:**
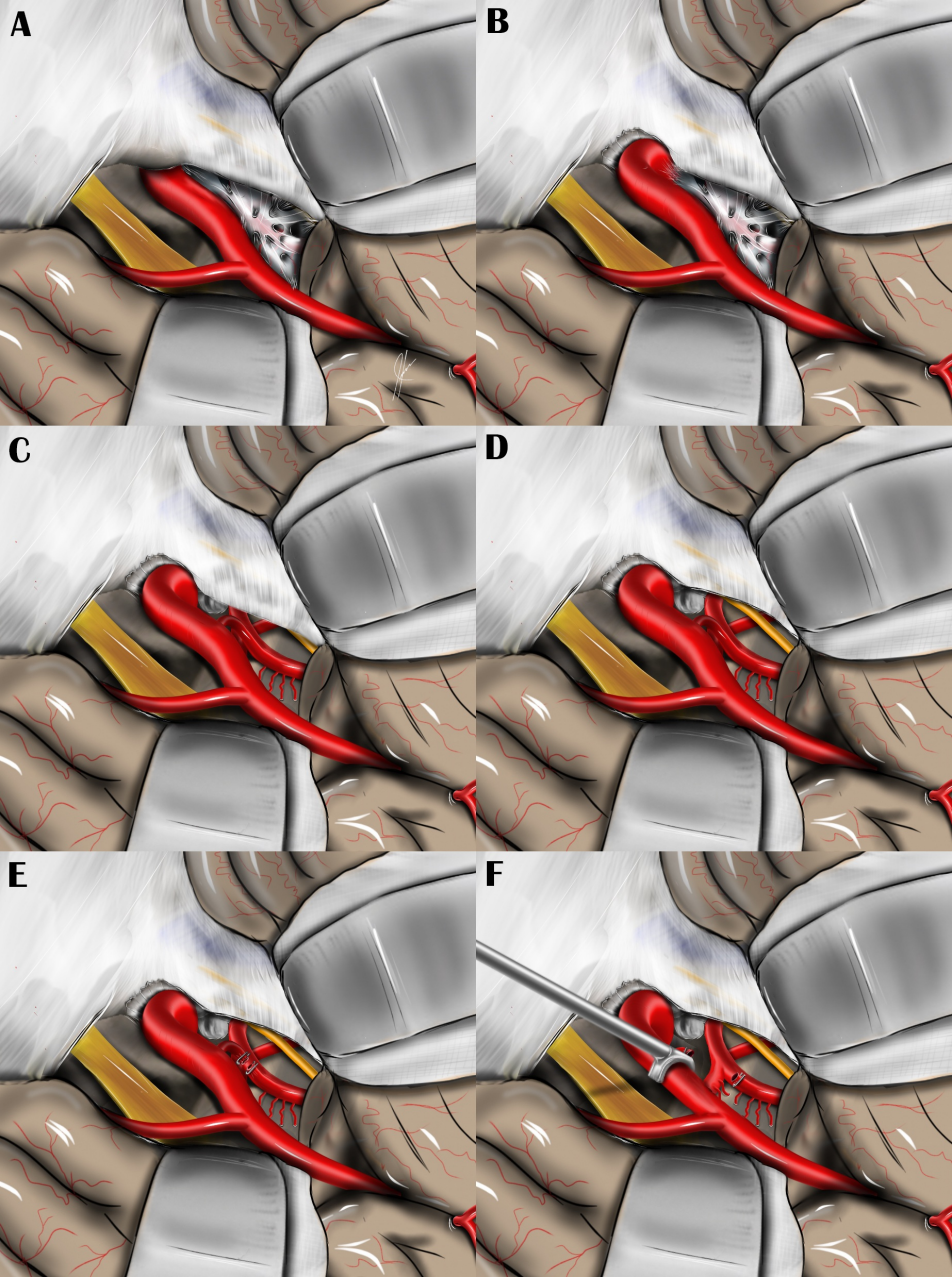
Steps of the transcavernous approach Artist's rendering of the modern surgical approach, with steps in the order they would be performed in the operating room, as illustrated with an artist’s rendering: A) before anterior clinoidectomy, B) after intradural anterior clinoidectomy, C) after posterior clinoidectomy, D) after cavernous sinus unroofing, E) after PCoA clipping and division, F) after ICA retraction.

First, the Sylvian fissure is widely split, distal to proximal, until the ICA is visualized (Figure 6A – ACP intact). Any adhesions between CN III and the uncus are dissected, allowing the temporal lobe to fall away from the cavernous sinus (CS).

At this point, if not performed extradurally, and felt necessary by the surgeon, an intradural anterior clinoidectomy is performed via a transverse dural incision with the high-power drill, following the same principles described above for an extradural clinoidectomy (Figure 6B). This may be followed with mobilization of the ICA with dissection of the distal dural ring, which facilitates later medial retraction.

Working lateral to the internal carotid artery and with the third nerve serving as a polestar, dissection is carried out until Liliequist’s membrane is reached, then opened. The posterior communicating artery (PCoA) is followed to its junction with the posterior cerebral artery (PCA), just anterior to CN III emerging from the pontomesencephalic junction. Liberating these critical neurovascular structures from their arachnoid adhesions leads to exposure of the posterior clinoid process (PCP). Intermittent medial retraction can then be applied to the ICA using a suction, allowing visualization of the dorsum sellae. Subsequently, a transverse dural incision is made, exposing the PCP, which is drilled down to a degree required to visualize the basilar apex, basilar trunk, the contralateral PCA, or pertinent pathology (Figure 6C).

For the namesake (transcavernous) portion of the procedure, an incision is made in the outer layer of the lateral wall of the CS along the course of the CN III within the oculomotor triangle as it enters the cavernous sinus. CN III can be observed passing into the lateral wall of the cavernous sinus through the lateral aspect of the oculomotor triangle—its borders formed by the dural adhesions between the petrous bone and anterior and posterior clinoid processes laterally and intraclinoid dural folds medially. When dissecting the dura in this region, care must be taken to avoid inadvertent injury to CN IV, which can occasionally cross over CN III above the ICA. By this point, the entire cisternal and cavernous segments of CN III have been well dissected. Next, CN IV is exposed from its point of entry into the lateral wall of the CS to the point where it crosses the ICA in the CS (Figure 6D). In some cases the dura between CN III and CN IV may be cut to allow lateral reflection of the tentorial edge and better visualization of the proximal basilar artery.

Having freed and exposed the optic nerve (ON), CN III, CN IV, and the distal intracavernous ICA, the remaining arachnoid and dural adhesions around these structures are sectioned, with particular attention paid to the distal dural ring. Next, the PCoA is noted emerging from the undersurface of the communicating segment of the ICA and travelling deep in the field towards the ipsilateral PCA. A perforator-free zone is then chosen as an appropriate spot to divide the artery. When isolated, titanium Weck clips (Teleflex Medical, Research Triangle Park, NC, USA) are placed and the artery sharply divided (Figure 6E). This maneuver allows a specialized vascular retractor (Samy Vascular Retractor, Integra Neurosciences, NJ, USA) to be placed on the intracranial ICA, widening the surgical line of sight to the upper retroclival space (Figure 6F).

Results

Each of the steps of the transcavernous approach incrementally widens the surgical window to the basilar apex. Posterior clinoidectomy afforded the lowest line of sight into the retrosellar region and provided the most “bang for the buck,” subjectively, as illustrated in Figure 4b. This step of the transcavernous approach appeared particularly well suited for viewing the lowest reaches of the retroclival region, which would otherwise be shielded by the PCP and upper clivus.

Cavernous sinus unroofing improved illumination of the operative field and increased lateral to medial working angles at the basilar apex (Figure 4d).

Anterior clinoidectomy allowed for a more anterior and midline surgical trajectory, which seemed ideal for pathology situated in or near the dorsal clival surface (Figure 4f).

**Figure 7:**
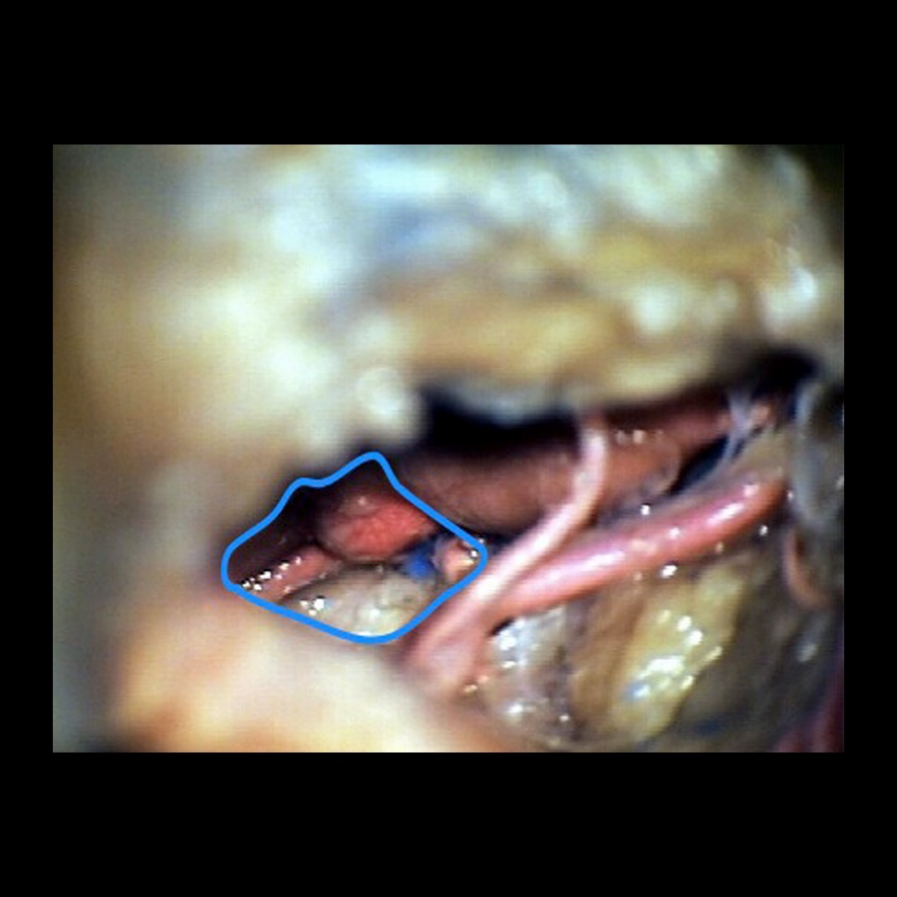
Microscopic view of the basilar apex View of bilateral P1 branches, basilar apex and aneurysm, and interpeduncular cistern revealed in the upper retrosellar region, as afforded by an orbitozygomatic craniotomy.

FTOZ was most beneficial in visualizing the upper retrosellar region, the interpeduncular cistern in particular, and the contralateral PCA. This additional line of sight can be contrasted with the more inferior line of sight afforded by the posterior clinoidectomy (Figure 7).

Illustrative case

History

A 37-year-old Caucasian female with a medical history of migraines and occipital neuralgia presented as a transfer from an outside hospital having developed a sudden onset, severe headache. Her family history was significant for a sister who underwent surgical resection of a cerebral arteriovenous malformation, as well as a cousin who underwent craniotomy and clipping of an intracranial aneurysm.

Examination

**Figure 8:**
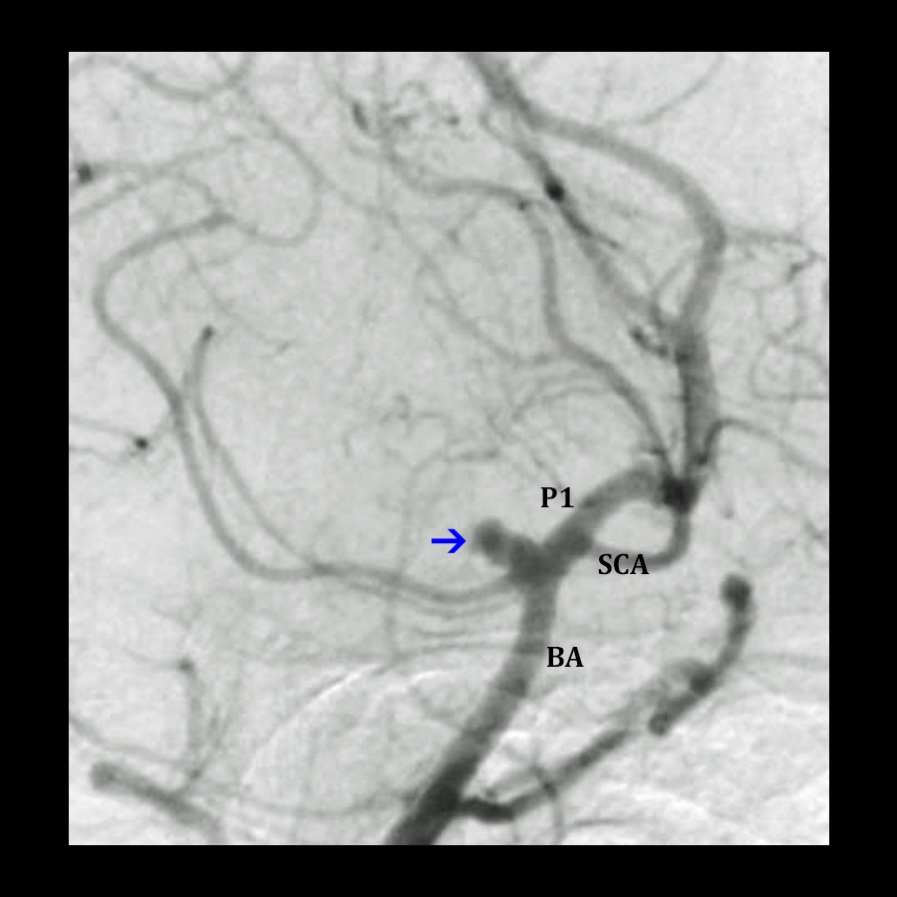
Angiography of a basilar apex aneurysm Digital subtraction angiography (lateral-oblique view) displaying ruptured basilar tip aneurysm. The aneurysm (blue arrow), posterior cerebral artery (P1), superior cerebellar artery (SCA), and basilar artery (BA) on the approached side are shown.

An outside hospital head CT revealed trace left Sylvian and basilar subarachnoid hemorrhage. On exam, the patient was neurologically intact. A diagnostic angiogram revealed a ruptured, wide-necked basilar tip aneurysm that the outside hospital neurointerventionalist did not deem suitable for coiling or stent-assisted coiling (Figure 8). The patient was transferred to our institution for evaluation for craniotomy and microsurgical clipping of the aneurysm.

Operation

**Figure 9:**
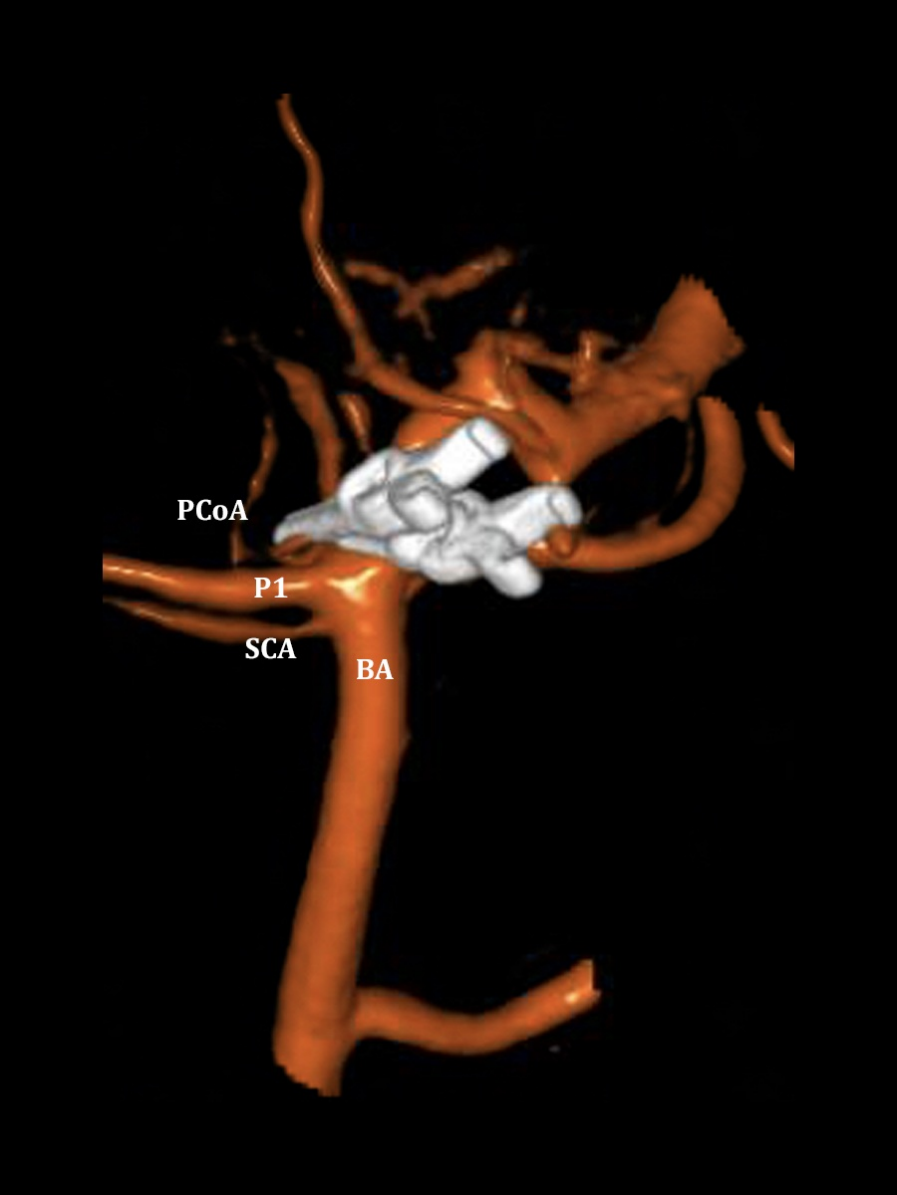
Postoperative 3D angiogram Dual volume three-dimensional rotational angiogram demonstrating complete occlusion of the previously noted basilar apex aneurysm following microsurgical clipping utilizing the transcavernous approach. The contralateral vessels are labeled for orientation: posterior communicating artery (PCoA), posterior cerebral artery (P1), superior cerebellar artery (SCA), and basilar artery (BA).

On the following day, the patient was taken to the operative room for craniotomy and aneurysm clipping. A left one-piece frontotemporal orbito-zygomatic craniotomy was performed, followed by an extradural anterior clinoidectomy, internal carotid artery retraction with the Samy vascular retractor, PCoA division, and posterior clinoidectomy. At this point the aneurysm was adequately visualized and cavernous sinus unroofing was deferred. The aneurysm was successfully obliterated with two aneurysm clips, one 5 mm mini clip across the aneurysm neck and a second 3 mm mini clip to address a small “dog-ear” neck remnant (Figure 9 and Video 1).

**Video 1:** Transcavernous approach to the basilar apex Diagnostic angiogram, operative approach, aneurysm dissection, and clipping via an orbitozygomatic craniotmy and transcavernous approach.

Hospital Course

The patient was admitted to the neurological intensive care unit postoperatively. She was neurologically stable throughout the remainder of her uncomplicated hospital course. On follow-up, the patient remains at her baseline, with some mild headache. Two-year follow-up angiography revealed complete occlusion of the aneurysm.

## Discussion

Each step of the transcavernous approach incrementally improves the illumination and visualization of, and working angles to, the basilar apex, while minimizing the need for brain retraction. Photographs taken of our cadaveric prosection provide powerful visual representation of the surgical views that can be created by each step.

Anatomy

An intimate knowledge of the anatomy is critical to the successful application of this approach, for which a brief review is offered. The interpeduncular cistern has an anterior border formed predominantly by the clivus and its superolateral extension, the PCP. The posterior border of the cistern is formed by the midbrain, specifically the cerebral peduncles and interpeduncular recess. The superior border of the cistern is formed by the mammillary bodies and the anterior perforating substance. No true inferior border exists, but one can be imagined caudally along the clivus at the line of transition from the interpeduncular cistern to the prepontine cistern as midbrain gives way to pons. The lateral border is formed by the mesial temporal lobe and the tentorium cerebelli, with CN IV coursing along its edge. The interpeduncular cistern is a midline structure and has no medial border, real or imagined.

The dominant vascular contents of the cistern are the bilateral superior cerebellar arteries (SCA), the basilar artery, and the bilateral P1 segments of the PCAs. Coursing inferiorly and medially from the communicating segment of the ICA in the paraclinoid region above, the PCoA traverses the interpeduncular cistern prior to joining the P1 segment. Arising from the PCoA are important thalamoperforators, which must be preserved during surgical exposure. Attention must also be paid to midbrain and thalamic perforators arising from the posterior aspect of the basilar apex and bilateral P1 segments, particularly when clipping basilar tip aneurysms. These perforators may often be hidden along the backside of such aneurysms, in a blind spot for the surgeon. This blind spot is minimized with the exposure afforded by the transcavernous approach.

Division of the PCoA may not be possible in all cases, notably in the presence of a fetal-origin PCA. Some surgeons contend that with sufficient dissection of the dural ring and dissection around CN III, the PCoA does not pose a significant barrier to retraction and visualization of the basilar apex.

Two important cranial nerves travel through the interpeduncular cistern en route to the cavernous sinus: the oculomotor nerve (CN III) and the trochlear nerve (CN IV). CN III arises from the anterior surface of the inferior extent of the midbrain and passes between P1 and SCA, then travels under the PCoA branch point from the ICA and enters the lateral wall of the cavernous sinus. CN IV arises from the posterior surface of the midbrain, just caudal to the inferior colliculus, wraps around the midbrain coursing anteriorly, and travels through the ambient cistern parallel to the free edge of the tentorium before crossing the lateral reaches of the interpeduncular cistern and entering the lateral wall of the cavernous sinus just below the PCP.

Surgical Exposure

Considering the limited natural corridors to the upper retroclival region and the critical nature of the neurovascular structures that stand between the surgeon and pathology in this area, maximal exposure is paramount. Every millimeter, literally, allows greater visualization and illumination. Previous anatomic studies have elucidated the value of various skull base approaches in creating safer surgical windows [14-16]. Likewise, the transcavernous approach expands the surgeon’s working corridor through the stepwise removal of bone and the untethering and mobilization of neural and vascular structures.

Though ample quantitative evidence suggests that skull base exposure increases angles of approach and area of exposure, in the case of the transcavernous approach, questions have remained about how beneficial each step is and if they are all necessary [14-17]. Each step is undoubtedly advantageous to some extent, but each also entails technically challenging dissection. Operative time is increased, and there is a greater risk of morbidity. In Dolenc’s original description of the approach, 3/11 patients had a poor outcome, one of which was a mortality [3]. Understanding which steps generate the best surgical corridors for a particular pathology may allow for a more streamlined approach that preserves the benefit of the transcavernous approach, while minimizing risk. The approach to each patient should be tailored, with only the necessary steps taken to safely access the basilar apex utilized.

Indications

Surgical intervention for basilar tip aneurysms, while safe and effective, is not without risk of morbidity and mortality and must have clear indications in the age of endovascular therapy. Even in expert hands, large series report surgical morbidity as high as 9% and permanent neurologic morbidity at 5% [10].

At our institution, challenging vascular lesions are discussed in an interdisciplinary neurovascular conference involving input from the Neurology, Neurosurgery, and Endovascular services. Two of the three endovascular faculty are dual fellowship-trained open vascular and endovascular neurosurgeons. While decision making for individual patients is made on a case-by-case basis, weighing a variety of factors including lesion anatomy and medical comorbidities, in general our indications to pursue open surgical treatment for basilar apex aneurysms include: 1) young age, 2) wide neck, 3) difficult endovascular access, 4) intracranial hemorrhage (subarachnoid or intraparenchymal) precluding use of anti-platelet agents, and 5) parent vessel incorporation in aneurysm dome.

As illustrated in this cadaveric prosection, some of the steps of the transcavernous approach yield particular advantages. The posterior clinoidectomy appears to yield the most generous improvement in deep surgical exposure and illumination, as well as affording the most inferiorly-directed surgical view, making it ideal for lower lying basilar apex aneurysms and retrosellar pathology. It is not routinely needed in all basilar apex aneurysms via the transcavernous approach. An important technical note is to identify the location of the posterior genu of the cavernous ICA, which may be in close proximity while completing the posterior clinoidectomy. Unroofing of the cavernous sinus further improves illumination and affords greater lateral to medial working room. Anterior clinoidectomy seems best suited for improving working angles directed at the clival surface in the anterior aspect of the retrosellar region. An orbitozygomatic craniotomy affords a more superiorly directed surgical view suitable for approaching pathology in the upper reaches of the retrosellar region, such as a high riding basilar apex aneurysm. Retraction of the ICA, as mentioned, carries the potential risk of retraction-induced spasm [18]. As such, we caution gentle retraction be used and that the artery is properly mobilized prior to that maneuver.

Limitations

The primary limitation of this study is its qualitative nature, inherent to its reliance on a single cadaver prosection. However, it is uncommon to encounter a cadaver harboring a basilar apex aneurysm, a unique finding that one might argue justifies our focus on a single specimen. Certainly, further cadaveric study utilizing multiple specimens could help to quantitatively validate our findings.

## Conclusions

The transcavernous approach, as initially described by Dolenc, is a useful technique for microsurgical clipping of basilar apex aneurysms. We explore the individual steps of the approach and their incremental additions to the exposure in a cadaver harboring such an aneurysm. An illustrative case and artist illustrations are used to emphasize the relative value of each step of the transcavernous exposure.
